# Phytochemical Composition and In Vitro Antibacterial Activity of the Essential Oil from *Lippia grata* Schauer (Verbenaceae) against *Staphylococcus* spp. from Caprine Mastitis

**DOI:** 10.1021/acsomega.5c06144

**Published:** 2025-09-29

**Authors:** Alisson T. da Silva, Danillo S. Rosa, Gutiele do N. do É, Lucas S. Azevedo, Ana V. V. de Souza, Márcio R. S. Tavares, Renata de F. S. Souza, Jackson R. G. da S. Almeida, Mateus M. da Costa

**Affiliations:** † 74373 Universidade Federal do Vale do São Francisco (UNIVASF), Petrolina, Pernambuco 56300-000, Brazil; ‡ 67744 Universidade Federal Rural de Pernambuco, Recife, Pernambuco 52171-900, Brazil; § Brazilian Agricultural Research Corporation (Embrapa) Semi-Arid, Petrolina, Pernambuco 56302-970, Brazil

## Abstract

*Staphylococcus* spp. are the primary pathogens responsible for caprine mastitis. These bacteria can form biofilms, hindering treatment and compromising milk production. In this context, plant-based natural products derived from *Lippia grata* Schauer have emerged as a potential alternative for treatment. This study aimed to evaluate the antibacterial and antibiofilm properties of the essential oil (EO) of *L. grata* against *Staphylococcus* spp. isolated from caprine mastitis. The EO was obtained by hydrodistillation, and its chemical constituents were identified by gas chromatography coupled with mass spectrometry. Fourteen clinical isolates of *Staphylococcus* spp. and two standard strains were tested. Antibacterial activity was assessed using broth microdilution. Biofilm production and the interference capacity of the EO were evaluated using a microplate adherence assay. The EO of *L. grata* yielded 5.47%, with carvacrol (78%), thymol (7.1%), and *p*-cymene (3.16%) as the main constituents. It exhibited inhibitory and bactericidal effects at concentrations ranging from 256 to 512 μg mL^–1^. All isolates formed biofilms, and the EO reduced biofilm formation in two isolates classified as moderate and strong producers. Additionally, it disrupted pre-established biofilms in all isolates. Therefore, the EO of *L. grata* exhibits antimicrobial and antibiofilm activities and represents a promising alternative for treating caprine mastitis, particularly in infections involving biofilm formation.

## Graphical abstract

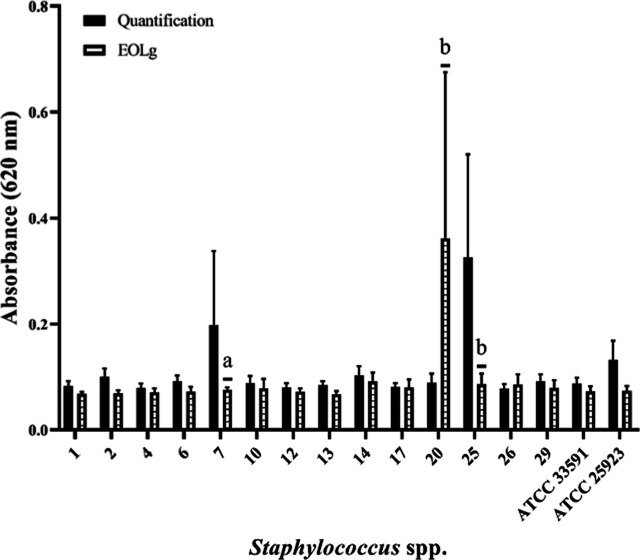

## 1 Introduction

Dairy goat farming in Brazil has experienced substantial growth over the past decade,^1^ with milk production estimated at approximately 300,000 tons in 2020.^2^ Within this context, the Northeast region—particularly the states of Bahia and Pernambuco—plays a prominent role. Together, these two states are home to 46% of the national herd and are responsible for 48.9% of milk production in the region and 34% of the overall output in the country.^3^

Milk productivity and quality are significantly impacted by the occurrence of caprine mastitis,^4^ defined as an inflammatory condition of the mammary gland that causes biochemical alterations in the milk and presents both local and systemic clinical symptoms.^5^ It is one of the most prevalent diseases in dairy goats and poses a considerable economic burden on rural properties.^6^ The combined effects of milk disposal and treatment expenses associated with the disease result in financial losses equivalent to approximately 36% of the total income of producers.^7^

*Staphylococcus* spp. are among the main causative agents of caprine mastitis.^8^ Foodborne illnesses caused by bacteria from this group pose a threat to public health^9^ due to the pathogenic components such as biofilm production and are frequently associated with resistance to multiple antibiotics.^10^ Biofilms are exopolymeric structures composed of carbohydrates, proteins, and DNA that surround the microorganisms.^11^ This matrix provides protection against immune responses, antimicrobial agents, and environmental conditions, while also facilitating the transfer of virulence genes between microorganisms.^12^ Consequently, infections involving biofilm formation often become chronic, leading to the excessive and recurrent use of antibiotics without clinical improvement, thereby contributing to increased pathogen resistance.^13^

Antibiotic therapy is the primary treatment for caprine mastitis.^14^ However, due to rising resistance, its efficacy has diminished.^10^ Therefore, the search for new alternative therapies has become critical. Historically, the plant kingdom has served as a rich source of chemical compounds for treating various diseases, including bacterial infections.^15^ Among plant-derived products, essential oils (EOs)—aromatic liquids containing complex mixtures of volatile and semivolatile phytochemicals produced via secondary metabolism—stand out.^16^ The literature has seen a growing number of studies evaluating the biological activity of EOs, especially regarding their antibacterial and antibiofilm potential, highlighting their relevance as promising sources for novel drugs.^17^

*Lippia grata* Schauer (Verbenaceae), a shrub species native to the Caatinga ecosystem in northeastern Brazil, its popular names are *alecrim-do-mato*, *alecrim-do-sertão*, or *alecrim-da-chapada* ^18^ and is mainly recognized for the medicinal properties of its EO.^19,20^ Studies have also reported the antibacterial potential of *L. grata* essential oil (EOLg).^21,22^ However, most of these studies have been conducted against standard bacterial strains or human clinical isolates, and little attention has been given to pathogens of veterinary relevance. To the best of our knowledge, no previous studies have evaluated the antibacterial and antibiofilm potential of *L. grata* EO against *Staphylococcus* spp. isolates obtained directly from cases of caprine mastitis. This gap is particularly relevant because it highlights the potential of this EO as a natural alternative strategy with great promise for sustainable animal production. Therefore, the aim of this research was to characterize the chemical profile and evaluate the in vitro antibacterial and antibiofilm activities of EOLg against *Staphylococcus* spp. strains isolated from caprine mastitis.

## 2 Materials and Methods

### 2.1 Collection of Plant Material

Leaves of *L. grata* were collected in March 2023, in the morning, at the Organic Medicinal Garden of the Federal institute of Sertão Pernambucano, Caatinga Biome in the region of Petrolina, Eastern Pernambuco State, Brazil (9°20′17.2″S and 40°41′56.4″W). Specimens were previously identified and deposited (no. 24998) in the Herbarium of Vale do São Francisco (HVASF).

Prior to collection, a voucher specimen was prepared, identified, deposited at the Herbarium of the Semi-Arid Tropics (HTSA—specimen no. 7232), and registered in SisGen under code AD0F005, in accordance with the Brazilian Biodiversity Law (13.123/2015). For the extraction of the essential oil, the leaves were air-dried at room temperature (≈28 °C ± 1 °C) for 7 days in the Biotechnology Laboratory of the same institution.

### 2.2 Extraction of EOLg

To obtain the EOLg, 100 g of dried *L. grata* leaves were subjected to hydrodistillation using a Clevenger-type apparatus for 4 h at 100 ± 5 °C. After the procedure, the essential oil yield was calculated, the aqueous phase was discarded, and the sample was stored in a freezer until further use.

### 2.3 Determination of the Chemical Composition of EOLg

The evaluation of EOLg constituents was performed by gas chromatography coupled with mass spectrometry and flame ionization detection (GC–MS/GC–FID) (GC-2010 Plus; GCMS-QP2010 Ultra, Shimadzu Corporation, Kyoto, Japan), equipped with an AOC-20i automatic sampler (Shimadzu). Separations were carried out using an Rtx-5MS Restek fused silica capillary column (5% diphenyl–95% dimethylpolysiloxane) measuring 30 m × 0.25 mm internal diameter (i.d.) and 0.25 μm film thickness, under constant helium flow (99.999%) at a rate of 1.2 mL min^–1^. The injection volume was 0.5 μL (5 mg mL^–1^), with a split ratio of 1:10. The oven temperature program started at 50 °C (isothermal for 1.5 min), followed by a 4 °C/min ramp up to 200 °C, then a 10 °C/min ramp up to 250 °C, with a final Isothermal hold for 5 min at 250 °C.

GC–MS and GC–FID data were simultaneously acquired using a detector splitting system with a flow split ratio of 4:1 (MS/FID). A 0.62 m × 0.15 mm i.d. capillary restrictor column connected the splitter to the MS detector, while a 0.74 m × 0.22 mm i.d. restrictor column connected the splitter to the FID detector. The injector temperature was set at 250 °C, and the ion source temperature at 200 °C. Ions were generated at 70 eV, with a scan rate of 0.3 scans s^–1^ across a mass range of 40–350 Da. The FID temperature was set at 250 °C, with synthetic air, hydrogen, and helium supplied at flow rates of 30, 300, and 30 mL min^–1^, respectively. The quantification of each constituent was estimated by normalization of the peak area obtained from the FID (%). Compound concentrations were calculated based on the GC peak areas and presented in order of GC elution.

Constituent identification was based on the comparison of retention indices with literature values. Retention indices were calculated using the van Den Dool and Kratz^23^ equation relative to a homologous series of *n*-alkanes (*n*C9–*n*C18). Three mass spectral libraries—WILEY8, NIST107, and NIST21—were used for spectral comparison, with an 80% similarity index as the threshold for identification.

### 2.4 Bacterial Isolates

A total of 14 clinical isolates of *Staphylococcus* spp. were selected from the microbial culture collection of the Laboratory of Animal Microbiology and Immunology, UNIVASF (SisGen A6C4D9C). These isolates were previously obtained from animals on six farms in the state of Pernambuco and one farm in the state of Bahia. Identification was performed using matrix-assisted laser desorption/ionization time-of-flight mass spectrometry (MALDI-TOF-MS), and the results are presented in Table 1 (unpublished data). As controls, two standard *Staphylococcus aureus* strains from the American Type Culture Collection (ATCC) were used: ATCC 33591, a methicillin-resistant *S. aureus* (MRSA), and ATCC 25923, a methicillin-sensitive *S. aureus* (MSSA) and biofilm formation control.

**Table 1:** Identification and Classification of *Staphylococcus* spp. Isolates from Caprine Mastitis on Brazilian Farms^a^

identification	property/state	species/MALDI-TOF
1	Juraci/Pernambuco	Staphylococcus chromogenes
2	Juraci/Pernambuco	S. chromogenes
4	Juraci/Pernambuco	S. chromogenes
6	Marciano/Pernambuco	S. aureus
7	Geraldo/Pernambuco	S. aureus
10	Clarice/Pernambuco	S. chromogenes
12	Clarice/Pernambuco	S. aureus
13	Ana Paula/Pernambuco	Staphylococcus xylosus
14	Ana Paula/Pernambuco	Staphylococcus caprae
17	Valberto/Pernambuco	S. aureus
20	Valberto/Pernambuco	Staphylococcus capitis
25	Domingos/Bahia	Staphylococcus lugdunensis
26	Domingos/Bahia	Staphylococcus simulans
29	Domingos/Bahia	Staphylococcus epidermidis
ATCC 33591	-	S. aureus
ATCC 25923	-	S. aureus

a - Data not collected. MALDI-TOF: matrix-assisted laser desorption/ionization time-of-flight mass spectrometry.

### 2.5 Essential Oil Solubilization

To prepare the stock solution, EOLg was dissolved in a dimethyl sulfoxide/sterile water mixture (15:85, v/v) to achieve a final concentration of 4096 μg mL^–1^, following the protocol described by Limaverde et al.^24^ After preparation, the solution remained in the dark and was kept at 8 °C prior to microbiological evaluations.

### 2.6 Antibacterial Activity

The minimum inhibitory concentration (MIC) and minimum bactericidal concentration (MBC) of EOLg were determined by broth microdilution, according to the guidelines of document M07.^25^ Assays were performed in 96-well microplates, with EOLg serially diluted in Mueller–Hinton (MH) broth in a 1:1 ratio, tested at concentrations of 2048, 1024, 512, 256, 128, 64, 32, and 16 μg mL^–1^.

To prepare the inoculum, freshly grown colonies on brain heart infusion (BHI) agar were suspended in 5 mL of 0.85% saline solution to a turbidity equivalent to 0.5 on the McFarland scale (1.5 × 10^8^ CFU mL^–1^). Then, 10 μL of this suspension was added to 990 μL of MH broth. From this dilution, 10 μL was inoculated into each well, and the plate was incubated at 37 °C for 24 h under aerobic conditions.

Subsequently, the content of each well was plated onto MH agar and reincubated at 37 °C for 24 h. The MBC was established as the lowest EOLg concentration able to eliminate the inoculum. Concurrently, the MIC was established as the lowest EOLg concentration able to inhibit bacterial growth, verified when the color did not change after adding 30 μL of 1% 2,3,5-triphenyltetrazolium chloride solution. All tests were conducted in technical and biological triplicate. Sterility and bacterial viability controls were also included.

### 2.7 Biofilm Quantification

The biofilm-forming phenotype of the isolates was evaluated using the microtiter plate adherence assay with modifications.^26,27^

Initially, isolated colonies were inoculated into 3 mL of Trypticase soy broth with 0.5% glucose (TSBg) and incubated at 37 °C for 24 h. Then, 195 μL of TSBg and 5 μL of the bacterial suspension (previously incubated) were added to each well of a microplate, followed by incubation at 37 °C for 24 h.

After incubation, 200 μL of sterile distilled water were used to clean each well and then the plate was air-dried for 5 min. The biofilm was fixed with methanol (150 μL in each well) at room temperature for 20 min, after which the solvent was removed and the plate stayed overnight at room temperature.

On the following day, the biofilm was stained with 100 μL of 0.25% crystal violet for 5 min, washed with sterile distilled water, and the remaining stain was solubilized with 200 μL of alcohol–acetone (80:20, v/v). Absorbance was assessed using a spectrophotometer (EXPERT PLUS-UV) at the wavelength of 620 nm. All assays were performed in technical and biological triplicate.

The isolates were classified in according to Stepanovic et al.,^27^ based on the optical density (OD) as follows: nonbiofilm producer (ODs < ODc), weak biofilm producer (ODc < ODs < 2 × ODc), moderate biofilm producer (2 × ODc < ODs < 4 × ODc), or strong biofilm producer (ODs > 4 × ODc), where ODs represents the optical density of the sample and ODc that of the negative control.

### 2.8 Antibiofilm Activity

The assay to evaluate the interference of essential oil with biofilm formation and on preformed biofilm in according to Merino et al.^26^ and Nostro et al.^28^ methods, with adaptations.

First, bacterial inocula were cultured in tubes containing 3 mL of TSBg at 37 °C for 24 h. Then, 100 μL of this culture was added to each well of a 96-well microplate along with 100 μL of the EOLg solution, resulting in a final concentration of 1/2 MIC. After incubation at 37 °C for 24 h, the microplate underwent similar to those of cleaning, fixation, dyeing, solubilization, and assessing procedures used in the biofilm quantification assay.

To assess the effect of EOLg on preformed biofilms, 5 μL of the bacterial inoculum in TSBg was added to wells containing 195 μL of TSBg and incubated at 37 °C for 24 h. The microplates were then washed three times with 200 μL of sterile distilled water and left to air-dry for 5 min. Finally, 200 μL of EOLg solution (at 1× and 1/2 MIC concentrations) was added. Optical density readings were taken immediately after the addition of EOLg (0 h) and after 24 h of incubation at 37 °C. The percentage of interference was determined using the formula: (mean OD_0_h/mean OD_24_h) × 100. All assays were performed in technical and biological triplicate on independent days.

### 2.9 Statistical Analysis

The biofilm interference results were analyzed using nonparametric multiple comparisons tests, equivalent two-way ANOVA with the posthoc test of Sidak. Statistical analyses were assessed by GraphPad Prism 8 software, and results were plotted as mean ± standard deviation. Only *p*-value <0.05 was considered significant.

## 3 Results and Discussion

### 3.1 Yield and Chemical Composition of EOLg

The yield and chemical composition of the essential oil from *L. grata* leaves, determined by GC–MS/GC–FID, are presented in Table 2. The yield of EOLg was 5.47% (v/w). A total of 21 components were identified, accounting for 97.9% of the total composition. The major constituents were carvacrol (78%), thymol (7.1%), and *p*-cymene (3.16%). Minor components included *E*-caryophyllene (1.56%), methyl thymol ether (1.39%), and γ-terpinene (1.35%).

**Table 2:** Chemical Composition of the Essential Oil from *Lippia grata* Schauer (Verbenaceae) Leaves

compound	IR^a^	%^b^
α-thujene	945	0.05
α-pinene	951	-
1-octen-3-ol	984	0.09
myrcene	994	0.49
α-terpinene	1020	0.25
***p*-cymene**	**1027**	**3.16**
1,8-cineole	1034	0.24
*E*-*b*-ocimene	1048	-
γ-terpinene	1061	1.35
*cis*-sabinene hydrate	1069	0.4
linalool	1102	0.53
ipsdienol	1148	0.61
terpinen-4-ol	1181	0.68
methyl thymol ether	1238	1.39
methyl carvacrol ether	1247	0.25
**thymol**	**1297**	**7.1**
**carvacrol**	**1311**	**78**
thymol acetate	1358	-
carvacrol acetate	1375	0.29
*E*-caryophyllene	1425	1.56
aromadendrene	1443	-
α-humulene	1458	0.26
ar-curcumene	1484	-
α-zingiberene	1496	-
*b*-bisabolene	1509	0.16
*d*-cadinene	1526	
spathulenol	1584	0.3
caryophyllene oxide	1591	0.76
humulene epoxide	1616	-
total detected (%)		97.9
yield % (v/p)		5.47

a Retention index calculated using the van Den Dool and Kratz^23^ equation in relation to a homologous series of *n*-alkanes (*n*C9–*n*C18).

b Compound content values obtained as the mean of three independent determinations by GC–MS and GC–FID. -: compound not detected.

The phytochemical profile of the *Lippia* genus has been confirmed in several studies, with variations in the proportions of components influenced by environmental factors such as soil composition, season, altitude, humidity, plant developmental stage, and collection time.^29–31^ One study observed greater chemical diversity in EOLg composition during the rainy season, highlighting the influence of seasonality on constituent concentrations and oil yield.^32^

Felix et al.^32^ identified thymol as the major compound across different seasons, with the highest concentration during the dry season. In contrast, *p*-cymene and carvacrol were not identified in samples collected in Ceará. Conversely, *p*-cymene, carvacrol, and γ-terpinene were the main constituents in EOLg extracted from plants collected in the states of Paraíba and Piauí.^31^ Essential oils typically exhibit a complex composition dominated by two or three major constituents, whose concentrations range from 20% to 70% compared to other components.^33^ The chemical similarity across different chemotypes suggests that the chemical profile of *Lippia* spp. essential oil is largely under genetic control, as the composition remains stable.^34^

The chemical profile of the EOLg obtained in this study showed carvacrol (78%) as the major component, accompanied by thymol and *p*-cymene in lower proportions. A previous study evaluating different doses of organic fertilization, with or without mineral supplementation, reported that nonirrigated plants fertilized with NPK exhibited increased levels of carvacrol.^35^ Almeida et al.^36^ also demonstrated that maintaining adequate levels of calcium, magnesium, and sulfur is essential for high carvacrol content in *L. grata*, whereas their deficiency significantly reduces this compound and increases the accumulation of *p*-cymene. High proportions of this compound (>40%) have also been reported in other *L. grata* chemotypes, reinforcing the consistency of our findings with chemical variations previously described in the species.^37–39^ It is worth noting that carvacrol, together with thymol, is widely recognized for its strong antimicrobial properties, including activity against *Staphylococcus* spp.^40^ Therefore, the predominance of carvacrol in our oil may be relevant for understanding its biological potential, while possible synergistic interactions with minor constituents cannot be excluded.

### 3.2 Antibacterial Activity

EOLg exhibited both inhibitory and bactericidal activity against all tested *Staphylococcus* spp. isolates, with MIC and MBC values ranging from 256 to 512 μg mL^–1^, including the ATCC 25923 and ATCC 33591 strains (Table 3).

**Table 3:** Antimicrobial Activity of the Essential Oil from *Lippia grata* Schauer (Verbenaceae) Leaves against *Staphylococcus* spp. Isolated from Caprine Mastitis^a^

	antimicrobial activity		classification of biofilm production
identification	MIC (μg mL^–1^)	MBC (μg mL^–1^)	classification
1	512	512	weak
2	512	512	weak
4	512	512	weak
6	256	256	weak
7	512	512	moderate
10	512	512	weak
12	512	512	weak
13	512	512	weak
14	512	512	weak
17	512	512	weak
20	256	256	weak
25	512	512	strong
26	256	256	weak
29	512	512	weak
ATCC 33591	256	256	weak
ATCC 25923	512	512	moderate

a ATCC: American Type Culture Collection; MIC: minimum inhibitory concentration; MBC: minimum bactericidal concentration.

Throughout the coevolutionary relationship between humans and plants, humankind has used the therapeutic properties of plants to treat infectious diseases. Among plant-derived products, essential oils—volatile compounds synthesized by various plant parts—have shown biotechnological potential for the development of new drugs targeting pathogenic microorganisms.^41^ The antimicrobial effect of plant-based compounds may be due to the action of a single secondary metabolite or the synergistic effect of multiple molecules, which can enhance inhibitory activity.^42^ Furthermore, these compounds may act on multiple bacterial targets, triggering different inhibitory mechanisms such as bacteriostatic or bactericidal effects.^43^ Another advantage of using EOs to treat microbial infections is their low cytotoxicity in mammals.^44^

In this context, essential oil extracted from *Lippia* spp. has demonstrated strong antimicrobial activity against Gram-positive pathogens.^45^ The antibacterial activity observed against *Staphylococcus* spp. isolates may be attributed to the major constituents present in EOLg.^46^ Literature reports indicate that thymol and carvacrol show antibacterial activity against *Staphylococcus* spp. isolated from bovine mastitis.^47^ Additionally, although *p*-cymene has limited antimicrobial activity on its own, it enhances the inhibitory effect when combined with carvacrol.^48^

Numerous studies have demonstrated the antimicrobial potential of thymol and carvacrol against Gram-positive bacteria, including *S. aureus*,^49^ *Streptococcus* spp.,^50,51^ and *Bacillus cereus*.^51^ The cytotoxic effect of the phenolic monoterpenoids thymol and carvacrol, as well as the monoterpene *p*-cymene, on microbial cells is due to increased permeability of the cytoplasmic membrane, leading to its rupture and subsequent leakage of intracellular content.^52,53^ The hydroxyl group in distinct positions on the aromatic ring of thymol and carvacrol is essential for their antimicrobial activity, enhancing hydrophilicity and reducing membrane potential.^53^ In contrast, *p*-cymene lacks a hydroxyl group; its antimicrobial effect results from accumulation in the membrane due to its affinity for liposomal membranes, causing membrane expansion and ion leakage.^52^

We did not perform direct comparisons with conventional antibiotics in the MIC/MBC assays, as the primary aim of this study was to assess the intrinsic antibacterial properties of EOLg. We acknowledge this limitation and emphasize that the susceptibility of the same isolates to antibiotics commonly used in veterinary clinical practice is being investigated in complementary studies by our group. Once available, these results will provide a better contextualization of the therapeutic potential of EOLg.

### 3.3 Biofilm

#### 3.3.1 Quantification of Biofilm Production

When evaluating the biofilm-forming ability of the clinical *Staphylococcus* spp. isolates on surfaces, all isolates (*n* = 14) demonstrated the ability to form biofilms (Table 1 and Figure 1). Among them, 85.71% (12/14) were classified as weak biofilm producers, whereas isolate 7 (1/14, 7.14%, *S. aureus*) was classified as a moderate producer and isolate 25 (1/14, 7.14%, *Staphylococcus lugdunensis*) as a strong producer.

**Figure 1:**
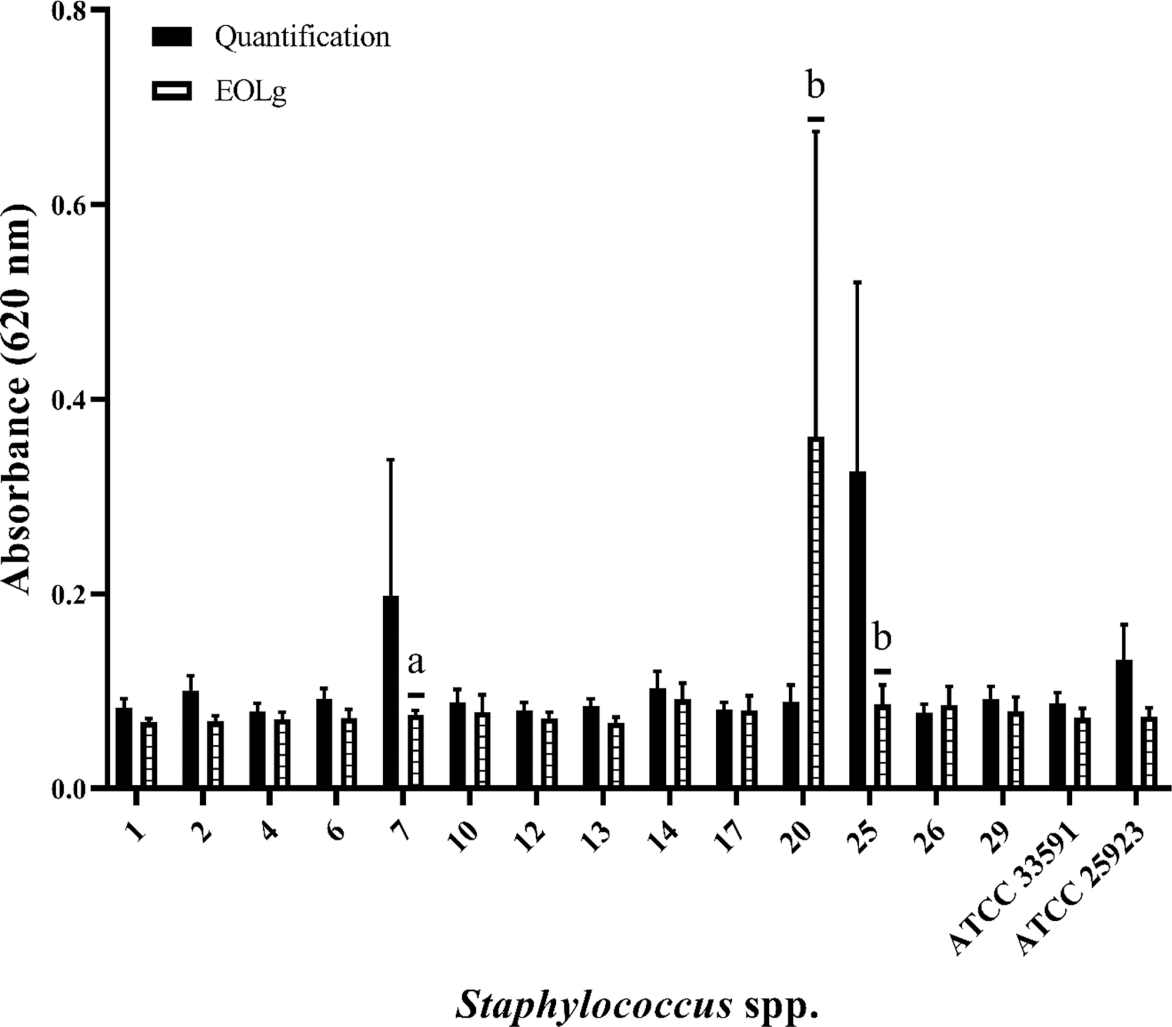
Effect of the essential oil from *Lippia grata* Schauer (Verbenaceae) leaves (EOLg) on the formation of biofilms produced by *Staphylococcus* spp. isolates from caprine mastitis. a: *p* < 0.01; b: *p* < 0.0001; ATCC: American Type Culture Collection; mean optical density of the negative control: 0.062.

*Staphylococcus* spp. strains isolated from mastitis cases are reported to be biofilm producers.^54^ This is a key virulence mechanism that protects bacteria and ensures their survival.^55^ Biofilm shields microorganisms from ultraviolet light and enhances resistance to extreme pH, high salinity environments, and the effects of antimicrobials.^56^ Furthermore, eradicating bacteria embedded in biofilms requires antimicrobial concentrations up to a thousand times higher than those needed to eliminate planktonic forms of the same bacteria, due to the physical and mechanical protection conferred by the biofilm matrix.^57^

#### 3.3.2 Antibiofilm Activity

As shown in Figure 1, the subinhibitory concentration of 1/2 MIC did not reduce biofilm formation in *Staphylococcus* spp. isolates classified as weak producers. However, for isolates 7 (*S. aureus*) and 25 (*S. lugdunensis*), EOLg was able to significantly reduce biofilm formation (*p* < 0.05). In contrast, isolate 20 (*Staphylococcus capitis*) exhibited increased biofilm formation when treated with EOLg (*p* < 0.0001). Additionally, concentrations of 1 and 1/2 MIC of EOLg were effective in disrupting preformed biofilms (Figure 2A,B).

**Figure 2:**
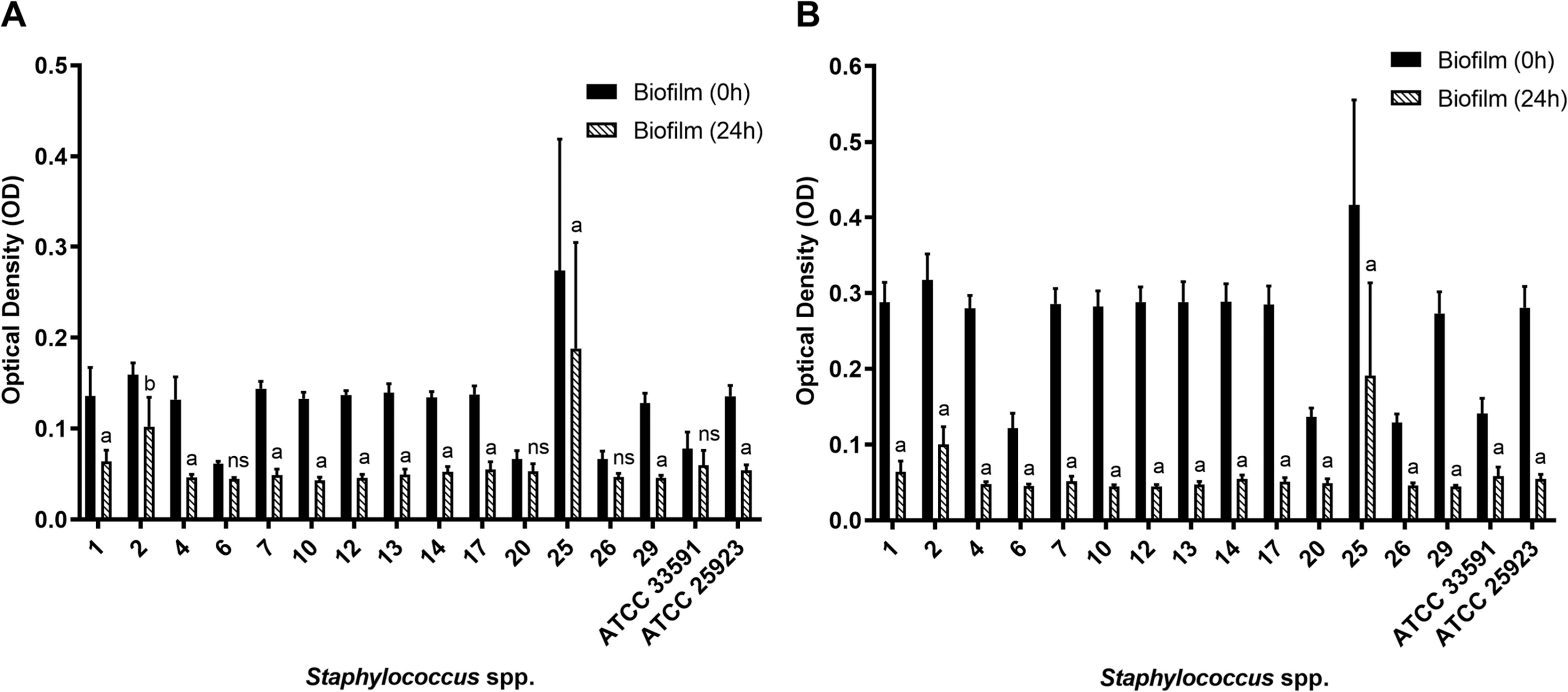
Effect of the essential oil from *Lippia grata* Schauer (Verbenaceae) leaves on pre-established biofilms produced by *Staphylococcus* spp. isolates. The essential oil concentrations tested were MIC (A) and 1/2 MIC (B). OD readings were taken immediately after exposure to the essential oil (0 h) and 24 h postapplication. a: *p* < 0.0001; b: *p* < 0.01; ns: not significant; ATCC: American Type Culture Collection.

Among the evaluation of the antibiofilm effects of *Lippia* spp. EO, some studies against both Gram-negative and Gram-positive bacteria stand out.^58,59^ Porfírio et al.,^58^ for example, evaluated the effect of essential oil extracted from the aerial parts of *Lippia alba* on biofilm formation by *S. aureus* (ATCC 6538). Among the three EO samples tested, the one with the highest activity inhibited biofilm formation by approximately 90% at a concentration of 500 μg mL^–1^ (1× MIC, 1× MBC). The major constituents identified in this oil were geranial (35.85%), neral (26.44%), and *p*-cymene (9.84%).^58^

Although most of the isolates evaluated in this study were classified as weak biofilm producers, the panel also included a moderate producer (*S. aureus* isolate 7), a strong producer (*S. lugdunensis* isolate 25), and the ATCC 25923 reference strain, which was also classified as a moderate producer. Similar findings were reported by Lopes et al.,^60^ in which EOLg inhibited biofilm formation in different *S. aureus* strains (including ATCC 25923) at concentrations ranging from 0.156 to 10 mg mL^–1^. In all these cases, EOLg demonstrated effectiveness both in preventing biofilm formation and in destabilizing pre-established biofilms, indicating that its activity was not limited to weak producers.

Interestingly, isolate 20 (*S. capitis*) showed an increase in biofilm formation when exposed to EOLg. This behavior may be related to a stress-adaptive response, since subinhibitory concentrations of antimicrobials are known to modulate pathways associated with biofilm formation in *Staphylococcus* spp. and other bacteria.^61,62^ Similar phenomena have been described as hormetic responses, in which low doses of stressors stimulate defense mechanisms, including the enhancement of biofilm production.^63^ Although the specific mechanism was not investigated in this study, it is important to elucidate the biochemical pathway of this phenomenon to better understand its implications for the therapeutic use of essential oils.

## 4 Conclusion

In conclusion, EOLg—whose main components were carvacrol, thymol, and *p*-cymene— exhibited antimicrobial and antibiofilm activity against *Staphylococcus* spp. isolates from caprine mastitis, and standard *S. aureus* strains resistant and susceptible to methicillin. These findings encourage further research on EOLg, particularly regarding its in vivo effects on caprine mastitis. However, we acknowledge that cytotoxicity assays were not conducted in this study. Since safety is a fundamental requirement for therapeutic application, additional studies are necessary to evaluate the cytotoxic and in vivo effects of this essential oil before its clinical or veterinary use can be proposed. Nevertheless, this work contributes to the growing body of knowledge on phytotherapy and alternative antimicrobials in veterinary medicine, providing relevant insights for the development of potential strategies to treat mastitis in dairy goat farming.

## Abbreviations

EO: essential oil
EOLg: essential oil of *Lippia grata*
DNA: DNA
GC–MS: gas chromatography coupled to mass spectrometry
GC–FID: gas chromatography coupled with flame ionization detection
UNIVASF: Universidade Federal do Vale do São Francisco
MALDI-TOF-MS: matrix-assisted laser desorption/ionization time-of-flight mass spectrometry
ATCC: American type culture collection
MIC: minimum inhibitory concentration
MBC: minimum bactericidal concentration
MH: Mueller Hinton
BHI: brain heart infusion
TSBg: trypticase soy broth with 0.5% glucose
OD: optical density
